# Professional Quality of Life Among Civilian Dentists During Military Conflicts: A Survey Study

**DOI:** 10.3390/healthcare13172155

**Published:** 2025-08-29

**Authors:** Yaniv Mayer, Maayan Atzmon Shavit, Eran Gabay, Thabet Asbi, Hadar Zigdon Giladi, Leon Bilder

**Affiliations:** 1 Department of Periodontology, School of Graduate Dentistry, Rambam Health Care Campus, Haifa 3525408, Israel; 2 Rappaport Faculty of Medicine, Technion Israel Institute of Technology, Haifa 3109601, Israel; 3 Independent Researcher, Haifa, Israel

**Keywords:** burnout, professional quality of life, dentists, armed conflict, anxiety, secondary traumatic stress, Israel, ProQOL, GAD-7, resilience

## Abstract

**Background**: Dental professionals are particularly susceptible to occupational stress and burnout, which are amplified during armed conflicts. Civilian dentists continuing to provide care under wartime conditions face unique psychological challenges. This study aimed to evaluate their psychological wellbeing and professional quality of life during military conflict. **Methods**: A cross-sectional study was conducted using an anonymous online questionnaire distributed through the national dental association. The survey included the Professional Quality of Life Scale (ProQOL, version 5) to assess compassion satisfaction, burnout, and secondary traumatic stress; and the Generalized Anxiety Disorder 7-item scale (GAD-7) to measure anxiety severity. Additional items captured demographic information, professional experience, pre-conflict workload, current work status, family circumstances, and subjective financial impact. The final sample included 239 civilian dentists. Statistical analysis included descriptive statistics, Pearson correlations, chi-square tests for categorical variables, Mann-Whitney U and Kruskal-Wallis tests for between-group comparisons, and multiple regression to identify predictors of psychological outcomes. **Results**: High compassion satisfaction was reported by 38.9% of respondents, while 70.3% exhibited average burnout levels; only 0.4% had high burnout. Secondary traumatic stress was low in 85.4% of participants. Minimal anxiety was found in 54% of respondents. Significant correlations were found between professional satisfaction and lower anxiety (*p* < 0.001), lower burnout (*p* < 0.001), and higher compassion satisfaction (*p* < 0.001). Dentists with more years of experience and older age reported lower anxiety and burnout levels. Higher pre-conflict workloads were associated with increased anxiety during the conflict (*p* < 0.001). Dentists working in Health Maintenance Organizations (HMOs) reported significantly higher anxiety levels compared to their non-HMO counterparts (*p* = 0.022), although reported income loss was similar between groups. **Conclusions**: Civilian dentists demonstrated resilience and overall positive professional functioning during prolonged conflict. However, public sector dentists, especially those in HMOs, showed greater vulnerability to anxiety. These findings underscore the need for systemic strategies to support dental professionals’ mental health during national crises, with emphasis on those in the public health system.

## 1. Background

Burnout among healthcare providers is a critical concern, affecting up to 50% of physcians and contributing to medical errors, reduced patient satisfaction, and mental health challenges [1,2,3]. Dentists experience comparable or higher prevalence rates (13–84%), influenced by job demands such as work overload, workplace conflicts, and the emotional demands of patient care [2,4,5,6,7]. Nangle et al. (2019) reported significantly higher burnout among dental professionals and students than in the general population [8], with doctor–patient interactions recognized as a major source of strain [9].

Armed conflicts amplify occupational stress, with healthcare professionals facing instability, trauma exposure, excessive workloads, and direct threats to themselves and their families [4,10,11,12,13]. A meta-analysis of medical teams in military operations found stress disorders at levels similar to those of combat soldiers, with longer exposure linked to greater psychological distress [13]. In conflict-affected regions, shortages of personnel and resources further increase vulnerability to burnout [4,12,13].

Since October 2023, armed conflict in the Middle East has posed unprecedented challenges to healthcare professionals in Israel, including civilian dentists, who have continued providing essential care during ongoing military activities. Although the effects of war on physicians and other medical staff are well documented [12,13], little is known about its impact on dentists, particularly in the Israeli context.

This study aimed to evaluate professional quality of life and psychological well-being specifically burnout, secondary traumatic stress, compassion satisfaction, and anxiety among Israeli civilian dentists during the ongoing military conflict that began on 7 October 2023.

## 2. Methods

### 2.1. Study Design, Participants, and Recruitment

A cross-sectional online survey was conducted in Israel following the outbreak of military conflict on 7 October 2023. The participants were Israeli civilian dentists who were actively engaged in clinical practice during the period of military activity. The study was conducted in accordance with the Declaration of Helsinki and approved by the Institutional Review Board (IRB) of Rambam Health Care Campus (approval number 0023-25). Informed consent was obtained from all subjects involved in the study. As the research involved no patient interaction and did not affect clinical care, it was exempt from full IRB review following assessment of the protocol and survey instruments.

There were no exclusion criteria on the basis of specialty, age, or practice setting to ensure a representative sample of the dental community.

The survey was distributed via an online platform by the Israeli Dental Association (IDA) to its nationwide membership via email, with a reminder sent two weeks later. It was also shared through professional WhatsApp groups for dentists. Participation was voluntary and anonymous; no identifying information such as names, email addresses, or IP addresses was collected at any stage. The survey was programmed to be accessible on various devices, including computers, tablets, and smartphones, to maximize accessibility and response rates. The survey began with an introductory statement outlining the study’s purpose, confidentiality measures, and the participants’ right to withdraw at any time without providing a reason. No incentives were offered.

### 2.2. Survey Instrument

The study’s questionnaire (Supplemental File S1) was based on the Professional Quality of Life Scale (ProQOL) [14] and the Generalized Anxiety Disorder 7-Item Scale (GAD-7) [15], both of which are validated and widely used instruments for assessing psychological outcomes among healthcare professionals. ProQOL assesses three core domains, namely, compassion satisfaction, burnout, and secondary traumatic stress, whereas the GAD-7 is a brief and reliable measure of generalized anxiety symptoms. Both tools have been previously applied in diverse healthcare contexts, including dental settings [4,6].

The questionnaire was structured into three sections:

1. Demographic and professional characteristics, including age, gender, years of experience, practice setting, and workload before and during the conflict, were recorded.
2. The ProQOL subscale is a 30-item self-report instrument designed to measure the positive and negative effects of working in helping professions [14]. It consists of three subscales:
  - Compassion Satisfaction (CS): the pleasure derived from being able to do one’s work well, reflecting professional fulfillment and meaning.
  - Burnout (BO): feelings of hopelessness, emotional exhaustion, and reduced efficacy, often stemming from chronic occupational stress.
  - Secondary traumatic stress (STS): symptoms related to indirect exposure to traumatic events through one’s work, including fear, sleep disturbances, and intrusive thoughts.

Each subscale includes 10 items rated on a 5-point Likert scale (1 = never to 5 = very often). ProQOL cutoffs for low, average, and high categories were applied as recommended in the official scoring manual by Stamm (2010), which is widely used in occupational health research. These thresholds are based on normative data and allow for standardized interpretation and comparison across studies [14].

3. The GAD-7 scale is a brief, 7-item questionnaire used to screen for generalized anxiety disorder and assess the severity of anxiety symptoms over the past two weeks [15,16,17]. The respondents rated how often they experienced symptoms such as nervousness, worry, restlessness, or irritability on a 4-point Likert scale (0 = not at all to 3 = nearly every day). The total scores range from 0–21 and are categorized as follows:
  - 0–4: minimal anxiety
  - 5–9: mild anxiety
  - 10–14: moderate anxiety
  - 15–21: severe anxiety

The ProQOL [11] and GAD-7 [17] questionnaires used in this study were validated Hebrew versions, which were previously translated and culturally adapted for use in Israeli healthcare settings. The demographic and professional characteristics questionnaires were developed in Hebrew, the primary language of most practicing dentists in Israel. Although no formal pilot testing was conducted for this study, all questionnaire tools, including the ProQOL and GAD-7, have been validated in both English and Hebrew and used successfully in prior research involving Israeli civilian dentists. Meyerson (2022) conducted a comprehensive analysis of stress coping strategies, burnout, secondary traumatic stress, and compassion satisfaction in this population under non-conflict conditions, confirming the instruments’ reliability and cultural relevance [4].

### 2.3. Sample Size Calculation

On the basis of previous research examining burnout and professional quality of life among healthcare professionals [14,15,16,17,18] and considering the estimated population of approximately 5000 dentists in Israel, a target sample size of at least 200 participants was established to achieve sufficient statistical power for the planned analyses. This sample size was calculated to provide 80% power to detect medium effect sizes at a significance level of α = 0.05.

### 2.4. Data Management and Security

All survey responses were automatically recorded in a secure database with restricted access. The data were exported to an Excel spreadsheet for cleaning and coding before being imported into statistical software for analysis. The database and all files containing study data were password protected and stored on secure servers accessible only to authorized research team members.

### 2.5. Statistical Analysis

Data analysis was conducted via IBM SPSS Statistics (Version 27.0, IBM Corp., Armonk, NY, USA). Descriptive statistics were computed for all variables: categorical data were summarized as frequencies and percentages, and continuous variables were summarized as the means and standard deviations. Professional quality of life (ProQOL) subscale scores were categorized into low, average, and high levels on the basis of established cutoff scores. Generalized anxiety disorder (GAD-7) scores were categorized as minimal (0–4), mild (5–9), moderate (10–14), or severe (15–21).

Pearson correlation coefficients were computed to evaluate the relationships between continuous psychological variables. Kruskal-Wallis tests were used to compare psychological outcomes among multiple groups according to age, years of professional experience, and anxiety severity, with significant findings further explored through pairwise comparisons via Bonferroni correction. Chi-square tests were used to examine associations between categorical variables, such as sex, specialist status, and war-related exposure, and psychological outcome categories. Mann-Whitney U tests were applied for comparisons between two groups where appropriate.

The relationships between preconflict working hours and psychological outcomes were evaluated via the Kruskal-Wallis test. Additionally, professional satisfaction scores were analyzed across psychological domains.

To assess differences in anxiety levels (GAD-7 scores) and reported income impacts between dentists working in health maintenance organizations (HMOs) and those who do not, the participants were divided into two groups: “working in HMO” and “not working in HMO”. A chi-square test was employed for comparisons regarding income impact, whereas a Mann-Whitney U test was utilized for comparing GAD-7 scores.

All the statistical tests were two-tailed, with a significance threshold set at *p* < 0.05. Missing data were managed via available case analysis, and the number of valid responses is indicated for each analysis.

## 3. Results

The study sample comprised 239 civilian dentists, of whom 60.3% were male, with a mean age of 50.7 years (SD = 14.3; range: 25–87). The majority were born in Israel (61.1%); most were married or partnered (79.9%) and reported having children (82.0%). Over half of the participants completed their dental education in Israel (51.5%), followed by eastern Europe countries (32.2%). In terms of professional experience, 39.0% had more than 31 years in practice, 20.8% had 11–20 years, and 15.7% had 21–30 years. Board-certified specialists represented 20.9% of the sample. The majority practiced private clinics (75.7%), with more than half also working in public health settings (56.9%) (Table 1).

### 3.1. War-Related Exposure, Professional Impact, and Work Patterns

In terms of work patterns, participants reported working an average of 36.6 ± 15.9 h per week prior to the conflict, which decreased to 33.0 ± 16.6 h during the war. This reduction was not statistically significant (*p* = 0.15). Despite the limited direct exposure, the conflict had a substantial impact on 49.4% of the participants’ professional functioning, and 93% experienced an actual reduction in earnings. The psychological effects on clinical decision-making were variable; 13% of the dentists indicated that the war influenced their own treatment planning, and 46% believed that these events affected their patients’ treatment decisions.

Notably, 71.5% of the respondents reported that, in retrospect, they would still choose dentistry as their profession.

### 3.2. Psychological Well-Being Measures

#### 3.2.1. Professional Quality of Life (ProQOL)

The majority of respondents reported high scores in the CS domain, indicating a strong sense of fulfillment and meaning in their professional roles. Responses in the burnout domain were more moderate, with many selecting mid-range scores, reflecting varying degrees of emotional exhaustion. In the secondary traumatic stress domain, the responses were more dispersed, with a subset of participants reporting higher scores suggestive of stress related to indirect exposure to trauma.

For the CS scale, 38.9% of the participants reported high satisfaction, 49.8% reported average levels, and 11.3% reported low satisfaction. The burnout scores were mostly within the average range (70.3%), with 29.3% reporting low burnout and only 0.4% reporting high burnout. The secondary traumatic stress scores were generally low: 85.4% fell in the low category, and 14.6% were in the average range; no participants scored in the high range.

#### 3.2.2. Anxiety Symptoms Scale (GAD-7)

According to the GAD-7 scores, 54.0% reported minimal anxiety, 29.7% reported mild, 11.3% reported moderate, and 5.0% reported severe symptoms.

#### 3.2.3. Professional Satisfaction and Psychological Outcomes

**Figure 1:**
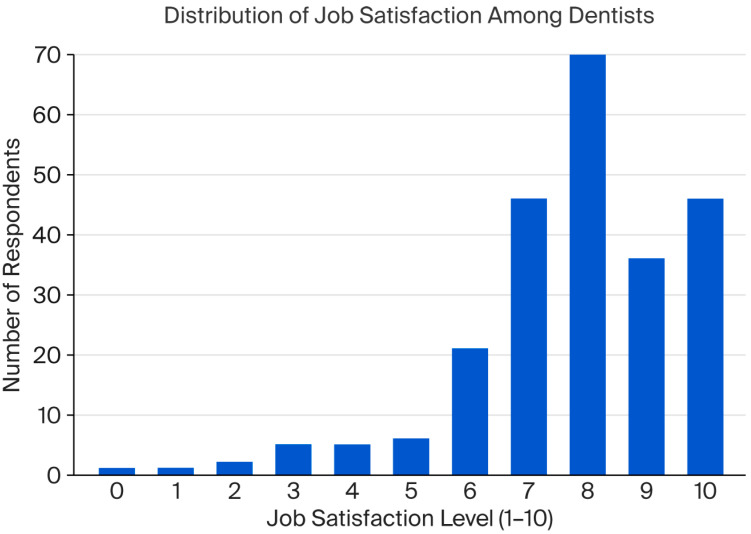
Distribution of job satisfaction among dentists.

Overall, satisfaction with the dental profession was generally positive, with a mean score of 7.8 out of 10 (SD = 1.8). Job satisfaction, measured on a scale from 1 (not satisfied at all) to 10 (very satisfied), showed that most participants reported high satisfaction: 29.3% selected a score of 8, 19.2% chose 10, and only 5.0% reported scores of 5 or below (Figure 1).

Higher satisfaction was significantly associated with more favorable psychological profiles, including average or high compassion satisfaction, low burnout, low secondary traumatic stress, and minimal anxiety (*p* < 0.001).

The participants with minimal anxiety reported significantly greater professional satisfaction than did those with moderate anxiety (*p* = 0.003) or severe anxiety (*p* = 0.009).

**Figure 2:**
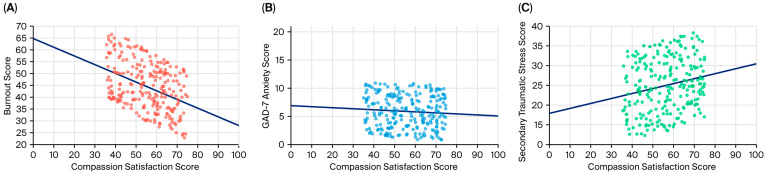
Correlations of compassion satisfaction with burnout (**A**), anxiety (**B**) and secondary traumatic stress (**C**).

These results indicated a significant negative correlation between compassion satisfaction and burnout (r = −0.517, *p* < 0.001) and between compassion satisfaction and GAD-7 scores (r = −0.128, *p* = 0.048). A weak but significant positive correlation was observed between compassion satisfaction and secondary traumatic stress (r = 0.196, *p* = 0.002). Higher levels of satisfaction with the dental profession were linked to average scores on the Compassion Satisfaction scale, lower levels of burnout and secondary traumatic stress, and minimal levels of anxiety as measured by the GAD-7. Furthermore, participants with minimal anxiety reported significantly greater professional satisfaction than did individuals with moderate (*p* = 0.003) or severe anxiety (*p* = 0.009) (Figure 2).

### 3.3. Demographic and Professional Associations

Older age and greater professional experience were significantly associated with lower burnout and anxiety levels. Compared with younger peers, dentists aged 54 years and older presented significantly lower burnout levels (*p* = 0.021), and those aged 53.5 years and older presented lower anxiety levels (*p* = 0.012). No significant associations were observed between age and compassion satisfaction or secondary traumatic stress. Similarly, more years of professional experience correlated with reduced burnout (*p* = 0.013) and anxiety (*p* = 0.011), without significant relationships with compassion satisfaction or secondary traumatic stress.

Gender was not significantly associated with any of the psychological outcomes, including compassion satisfaction (*p* = 0.33), burnout (*p* = 0.44), secondary traumatic stress (*p* = 0.47), or anxiety (*p* = 0.17). Furthermore, no significant differences were found between general practitioners and board-certified specialists across any of the psychological domains.

**Figure 3:**
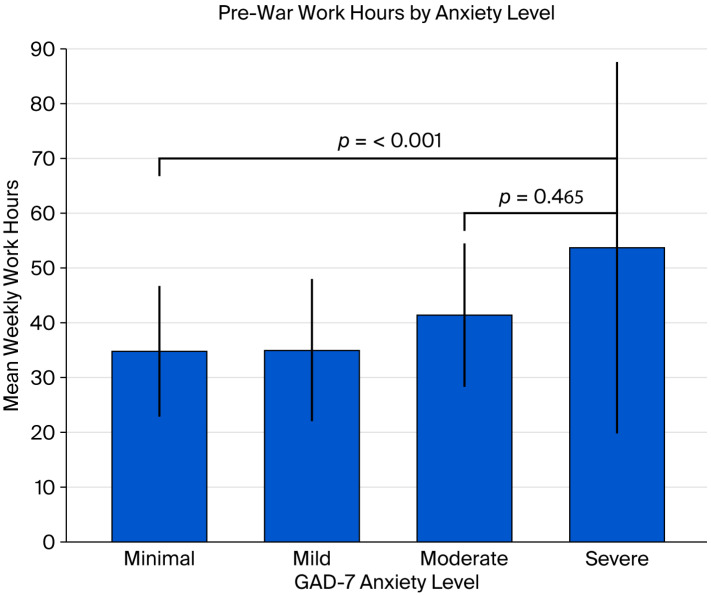
Higher anxiety levels were associated with longer prewar working hours, with the severe anxiety group averaging over 53 h per week.

Preconflict workload was significantly associated with psychological outcomes. Compared with those with low secondary traumatic stress, those with average secondary traumatic stress reported greater weekly working hours prior to the conflict (41.9 vs. 35.7 h; *p* = 0.033). Additionally, participants with severe anxiety symptoms had worked significantly more hours before conflict (mean = 53.7 h per week), indicating a strong relationship between a greater workload and elevated anxiety levels (*p* < 0.001) (Figure 3).

Compared with those not working in HMOs, those working in HMOs reported significantly higher anxiety levels (median GAD-7 scores) (Mann-Whitney U = 8204.0, *p* = 0.022). However, there was no statistically significant difference in reported income impact between these two groups (χ^2^ = 2.18, *p* = 0.14).

## 4. Discussion

This study evaluated the professional quality of life among civilian dentists during an ongoing military conflict and demonstrated generally positive outcomes despite substantial conflict-related stressors. A considerable proportion of dentists reported high compassion satisfaction (38.9%), average burnout levels (70.3%), and minimal secondary traumatic stress (85.4%). Anxiety levels were predominantly low, correlated negatively with burnout and positively with compassion satisfaction, underscoring significant resilience among dental professionals in challenging environments.

Burnout, characterized by emotional exhaustion, depersonalization, and diminished personal accomplishment, significantly affects healthcare providers by reducing job satisfaction, performance, and interpersonal relationships and increasing vulnerability to illness [19]. Professional quality of life (ProQOL), encompassing compassion satisfaction, burnout, and secondary traumatic stress, provides a comprehensive framework for evaluating dentists’ experiences, particularly under stress [20].

The current findings align with previous research demonstrating varied responses to stress among healthcare professionals, which are influenced by protective and risk factors. Meyerson et al. similarly reported high compassion satisfaction and moderate burnout among civilian dentists, attributing these outcomes to effective coping mechanisms and resilience [4]. Additionally, Long et al. highlighted substantial variability in burnout prevalence (13–84%) among dentists internationally, which was influenced by local contexts, occupational demands, and available resources [7].

In contrast to findings from other conflict-affected regions, such as elevated burnout levels reported among Palestinian healthcare workers due to chronic stress and limited coping resources [21], this study revealed comparatively lower levels of burnout and secondary traumatic stress among civilian dentists. This discrepancy likely reflects unique protective factors within the Israeli context. Factors such as older age and greater professional experience were significantly associated with lower burnout and anxiety, which is consistent with the literature emphasizing the importance of experience in building resilience and adaptive coping strategies among healthcare workers [12,13]. Robust familial and social support networks, previously identified as critical protective factors against burnout [22,23], likely mitigated psychological distress in this population. Spousal and familial support was particularly essential, given additional stressors from family members’ reserve duties and related work burdens.

Despite stressful conditions, dentists expressed considerable professional satisfaction and reaffirmed their choice of profession, aligning with broader evidence suggesting that healthcare providers maintain professional fulfillment despite occupational stressors due to intrinsic rewards, meaningful patient interactions, and professional accomplishments [24,25].

The lack of a statistically significant change in working hours suggests either substantial variability in responses or that some dentists were able to maintain their schedules despite the conflict, possibly reflecting adaptive strategies or differing levels of exposure.

Financial pressures, exacerbated by family members’ deployment and reliance on a single income, presented additional economic burdens for dentists. Financial stress among healthcare professionals, particularly physicians, significantly contributes to burnout and decreased job satisfaction, particularly in settings of increased workload and reduced financial security [26,27]. Although no statistically significant difference was observed regarding income impact between dentists working in health maintenance organizations (HMOs) and those who did not, anxiety levels were significantly higher among those affiliated with HMOs. This heightened anxiety potentially reflects greater exposure to administrative stressors and workload demands typical of public healthcare settings, as documented in studies of physicians working in HMOs and similar organizations [28,29].

Dentists face additional complexities in treating patients experiencing trauma or displacement, paralleling professional challenges reported among healthcare providers during conflicts elsewhere, such as Ukraine and other war-affected regions [30]. These responsibilities elevated psychological distress, highlighting the universality of such challenges during conflicts.

Consistent with broader research, this study revealed a notable correlation between preconflict workloads and anxiety during conflict, suggesting that heavier workloads exacerbate anxiety and burnout during crises [6,7]. These findings emphasize the critical need for systemic interventions targeting workload management and professional support during prolonged stress periods.

Cultural factors unique to the Israeli context may influence both the expression and reporting of occupational stress and satisfaction. For example, the prevalence of social solidarity, collectivism, and norms surrounding resilience in times of crisis could modulate psychological responses among dentists. Additionally, attitudes toward mental health, help-seeking behavior, and professional support structures may shape how stress and satisfaction are perceived and addressed in the Israeli dental profession [31,32].

Israeli healthcare professionals are often exposed to recurrent periods of national stress, which can foster adaptive coping strategies but may also contribute to chronic occupational strain. This context differs from that of dentists in other countries facing systemic stressors, such as the COVID-19 pandemic. For example, during COVID-19, Polish and Turkish dentists experienced elevated anxiety levels and changes in working conditions, but with notable variations linked to local policy responses and healthcare infrastructure. Polish dentists reported lower mean anxiety than their Turkish counterparts during the pandemic but experienced more pronounced increases in state anxiety, possibly due to a higher infection rate and fewer lockdowns in dental practice. Differences in fear and anxiety were also associated with factors such as risk of infection, government support, and access to personal protective equipment. These cross-national findings highlight how cultural context, systemic structures, and crisis type shape the mental health impacts experienced by dental professionals in times of adversity [33].

In addition to their clinical implications, these findings hold significant relevance for health policy. In Israel, public dental care under the National Health Insurance Law covers critical populations, including children and elderly individuals. Ensuring psychological resilience among dental providers during military conflicts is essential for maintaining stable service delivery and protecting vulnerable populations’ welfare. Policymakers must prioritize healthcare professionals’ mental health when planning services during national crises. In light of these findings, targeted support for public sector dentists during crises is warranted. Potential measures include flexible work scheduling, provision of mental health services, structured peer-support programs, and temporary financial assistance. Establishing clear crisis protocols and ensuring availability of protective resources could help maintain both professional functioning and patient care quality.

While this study provides valuable insights into the professional quality of life of civilian dentists during an ongoing military conflict, the absence of a control group limits the ability to directly compare these findings to pre-conflict conditions or to unaffected populations. Establishing a “clean” pre- and post-conflict comparison is inherently challenging in such dynamic and unpredictable circumstances, as baseline data are often unavailable and the onset of conflict is sudden. Nevertheless, future research could benefit from longitudinal designs or opportunistic use of pre-existing datasets to capture changes over time. Comparative studies involving healthcare professionals in regions not affected by conflict, or those experiencing different types of crises, may also help contextualize the impact of armed conflict on quality of life. Moreover, given the well-documented negative effects of war on psychological well-being, further studies should examine targeted interventions such as structured peer-support programs, workload redistribution strategies, and resilience training that may mitigate these effects and support the mental health of dental professionals in conflict zones.

This study has several limitations that should be acknowledged. The cross-sectional design precludes establishing causality and tracking temporal changes in the measured outcomes. Additionally, the lack of a control group prevents meaningful comparisons with either pre-conflict baseline data or unaffected populations, a limitation that stems from the logistical and ethical challenges inherent in wartime research, where sudden conflict onset and persistent instability make baseline data collection and longitudinal follow-up particularly difficult. The study’s reliance on self-reported measures may introduce recall and social desirability biases, while the potential underrepresentation of certain dental practitioner subgroups despite nationwide recruitment efforts may affect the generalizability of findings. In addition, the association between higher anxiety and longer pre-conflict working hours observed in this study appears to be unrelated to the war itself, and may instead reflect the absence of standardized working hours among dentists, which could have influenced baseline anxiety levels.

The results specifically reflect experiences within the Israeli healthcare system during military conflict and may have limited applicability to dental professionals in other contexts due to differences in healthcare infrastructure, social support networks, and conflict characteristics. Future investigations should prioritize prospective, multi-center, and controlled methodologies when possible, focus on developing interventions to mitigate conflict’s impact on healthcare providers’ well-being, and conduct comparative studies across diverse populations and settings to better establish the broader relevance of these findings.

## 5. Conclusions

Despite its limitations, this study offers important insights into the resilience and psychological well-being of civilian dentists working under military conflict stressors. It identifies protective factors and calls for targeted measures such as workload management, stronger social support systems, and resilience building initiatives to preserve mental health and professional quality of life. The results emphasize the need for health authorities to implement such strategies during prolonged crises.

## Figures and Tables

**Table 1:** Participant demographic and professional characteristics (n = 239).

Variable	Category	n (%)
Gender	Male Female	144 (60.3%) 95 (39.7%)
Age	Mean ± SD (range)	50.7 ± 14.3 (25–87)
Country of Birth	Israel Eastern Europe/FSU (incl. Romania) Other	146 (61.1%) 63 (26.4%) 30 (12.5%)
Marital Status	Married/Partnered	191 (79.9%)
Has Children	Yes	196 (82.0%)
Religion/Ethnicity	Jewish Arab Other	204 (85.4%) 29 (12.1%) 6 (2.5%)
Country of Dental Education	Israel Romania Russia and FSU Jordan France Argentina Hungary Italy USA Mexico Slovakia Other	123 (51.5%) 40 (16.7%) 37 (15.5%) 8 (3.3%) 5 (2.1%) 4 (1.7%) 3 (1.3%) 2 (0.8%) 2 (0.8%) 2 (0.8%) 2 (0.8%) 11 (4.6%)
Years of Professional Experience	Mean ± SD (range) 1 year 2–5 years 6–10 years 11–20 years 21–30 years 31+ years	23.3 ± 14.3 (1–58) 7 (3.0%) 28 (11.9%) 23 (9.7%) 49 (20.8%) 37 (15.7%) 92 (39.0%)
Board-Certified Specialist	Yes	50 (20.9%)
Specialty (among specialists)	Oral & Maxillofacial Surgery Prosthodontics Orthodontics Endodontics Pediatric Dentistry Periodontology Other	8 (16.0%) 11 (22.0%) 5 (10.0%) 7 (14.0%) 4 (8.0%) 7 (14.0%) 8 (16.0%)
Practice Setting	Private Public (HMO) Corporate/Managed Care Other	181 (75.7%) 136 (56.9%) 22 (9.2%) 27 (11.3%)

FSU—former Soviet Union countries.

## Data Availability

The data presented in this study are available on request from the corresponding author.

## Supplementary Materials

The following supporting information can be downloaded at: https://www.mdpi.com/article/10.3390/healthcare13172155/s1, File S1: GAD-7 Anxiety.

## Abbreviations

ProQOL	Professional Quality of Life
GAD-7	Generalized Anxiety Disorder 7-Item Scale
CS	compassion satisfaction
BO	Burnout
STS	Secondary Traumatic Stress
IDA	Israeli Dental Association
IRB	Institutional Review Board
SPSS	Statistical Package for the Social Sciences
FSU	Former Soviet Union
HMO	Health maintenance organization
