# Supplementary material for: Professional Quality of Life Among Civilian Dentists During Military Conflicts: A Survey Study
Source: Healthcare (Basel). 2025 Aug 29;13(17):2155. doi: 10.3390/healthcare13172155 (PMC12428038; doi:10.3390/healthcare13172155)
Supplement: Supplementary file 1 [file healthcare-13-02155-s001.zip › healthcare-3795605-supplementary.pdf]

GAD-7 Anxiety

Over the last two weeks, how often have you been bothered by the following problems?	Not at all	Several days	More than half the days	Nearly every day
1. Feeling nervous, anxious, or on edge	0	1	2	3
2. Not being able to stop or control worrying	0	1	2	3
3. Worrying too much about different things	0	1	2	3
4. Trouble relaxing	0	1	2	3
5. Being so restless that it is hard to sit still	0	1	2	3
6. Becoming easily annoyed or irritable	0	1	2	3
7. Feeling afraid, as if something awful might happen	0	1	2	3

Column totals _____ + _____ + _____ + _____ =

Total score ________

If you checked any problems, how difficult have they made it for you to do your work, take care of things at home, or get along with other people?

Not difficult at all	Somewhat difficult	Very difficult	Extremely difficult
☐	☐	☐	☐

Source: Primary Care Evaluation of Mental Disorders Patient Health Questionnaire (PRIME-MD-PHQ). The PHQ was developed by Drs. Robert L. Spitzer, Janet B.W. Williams, Kurt Kroenke, and colleagues. For research information, contact Dr. Spitzer at ris8@columbia.edu. PRIME-MD® is a trademark of Pfizer Inc. Copyright© 1999 Pfizer Inc. All rights reserved. Reproduced with permission

Scoring GAD-7 Anxiety Severity

This is calculated by assigning scores of 0, 1, 2, and 3 to the response categories, respectively, of “not at all,” “several days,” “more than half the days,” and “nearly every day.”
GAD-7 total score for the seven items ranges from 0 to 21.

0–4: minimal anxiety

5–9: mild anxiety

10–14: moderate anxiety

15–21: severe anxiety
